# Assessment of comorbidity awareness in patients with atrial fibrillation: The ACAPAF study

**DOI:** 10.1016/j.ijcha.2025.101813

**Published:** 2025-10-02

**Authors:** Rana Önder, Lien Desteghe, Johan Vijgen, Hein Heidbuchel

**Affiliations:** a Faculty of Medicine and Life Sciences, Hasselt University, Martelarenlaan 42, 3500 Hasselt, Belgium; b Heart Centre Hasselt, Jessa Hospital, Stadsomvaart 11, 3500 Hasselt, Belgium; c Department of Cardiology, Antwerp University Hospital, Drie Eikenstraat 655, 2650 Edegem, Belgium; d Centre for Research and Innovation in Care (CRIC), Department of Nursing and Midwifery Sciences, University of Antwerp, Prinsstraat 13, 2000 Antwerp, Belgium; e Research Group Cardiovascular Diseases, GENCOR, University of Antwerp, Prinsstraat 13, 2000 Antwerp, Belgium

**Keywords:** Atrial fibrillation, Comorbidities, Awareness, Care pathways, EHRA-PATHS

## Abstract

**Background and aim:**

Systematic and integrated comorbidity management in patients with atrial fibrillation (AF) requires patient involvement, starting with comorbidity awareness. This study evaluates comorbidity awareness in patients with AF before and after a first nurse-led AF clinic visit and after six months. We also measured the time needed for full comorbidity mapping using the EHRA-PATHS software.

**Methods:**

This prospective two-centre study included patients diagnosed with AF attending the AF clinic for the first time. The software systematically assessed 23 comorbidities. Patients completed a comorbidity awareness questionnaire, focusing on nine AF-related comorbidities two weeks before their first visit, less than a week after, and six months later. Patients also had a telephone consultation with the AF nurse to discuss their comorbidities 1–3 months post-visit.

**Results:**

The study included 76 patients (mean age 68.3 ± 10.3 y). Baseline awareness of comorbidity relevance for AF ranged between 11.1–100.0 %. Awareness about own alcohol consumption was the most ‘underestimated’ comorbidity before first contact (50.0 %), while smoking was most ‘overestimated’ (55.6 %; i.e. admitted in private but not during formal evaluation by nurses). The impact of an AF clinic visit on awareness of personal comorbidities was limited (p = 0.456), and also after an additional phone consultation, awareness was suboptimal after six months (p = 0.099). AF nurses needed 18.4 ± 8.7 min to complete the software.

**Conclusions:**

Patients’ comorbidity awareness is moderate, and more educational efforts are needed to improve their awareness. A systematic and complete comorbidity evaluation at the AF clinic using EHRA-PATHS software can be done within a reasonable time frame.

## Highlights

• Diabetes is a well-recognised comorbidity by patients with AF.
• Alcohol consumption and physical inactivity are often underestimated and smoking underadmitted to the health care workers.
• Patients’ comorbidity awareness is moderate.
• More educational efforts than a single in-person session and additional phone consultation are needed for improve awareness.
• The EHRA-PATHS software is a feasible tool for comorbidity evaluation in patients with AF within a reasonable time frame.

## Graphical abstract

**Figure:**
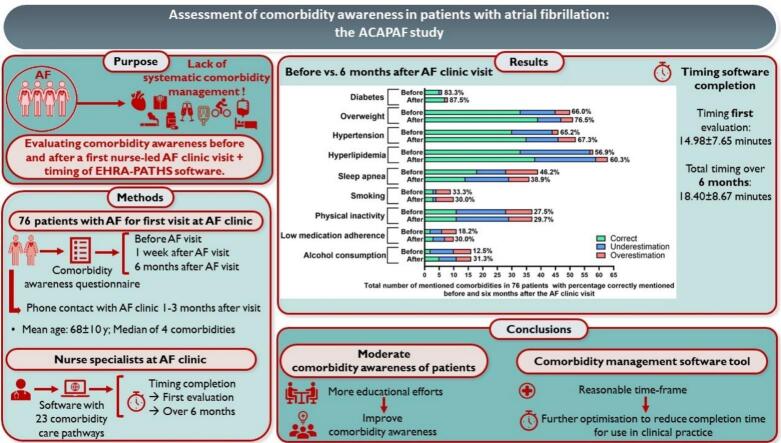

## 1 Introduction

Patients with atrial fibrillation (AF) over age 65 years have an average of five comorbidities [[1], [2], [3], [4]]. Multimorbidity, which refers to the presence of two or more concomitant health conditions, is associated with worse outcomes [5,6]. The most common comorbidities in patients with AF are hypertension, heart failure, hyperlipidemia, coronary artery disease, overweight, chronic kidney disease, and diabetes. [1,[6], [7], [8], [9]]. The primary component of the AF-CARE approach of the 2024 European Society of Cardiology guidelines for AF management is addressing comorbidities and risk factors [10]. However, previous studies have demonstrated a lack of systematic and integrated comorbidity management in patients with AF in current clinical practice [11,12]. Moreover, since patient involvement is important for comorbidity management, this starts with their comorbidity awareness. However, there is indication that patients have limited awareness of their comorbidities [[13], [14], [15]].

The international European Horizon 2020 project EHRA-PATHS (grant nr. 945260) developed a software tool to detect and manage 23 comorbidities in patients with AF [[16], [17], [18]]. That newly developed software tool was used in this study to systematically map the comorbidity profile of patients with AF, serving as the ground truth. The aim of this prospective study was to evaluate the awareness of the patients about AF comorbidities in general, and their own comorbidities in particular, both before and after a first nurse-led AF clinic visit, and after six months. However, distinguishing between patient knowledge (i.e., a comprehensive understanding of the condition and its management) and patient awareness (i.e., a more general recognition of the condition and its comorbidities) is difficult in this setting. We also measured the time needed for the full comorbidity mapping, and comorbidity management initiation, with the help of the EHRA-PATHS software tool.

## 2 Methods

### 2.1 Study design and study population

This prospective, two-centre, non-randomised study was performed at two large tertiary care centres, i.e. the Antwerp University Hospital (UZA) and Jessa Hospital Hasselt (Jessa). Consecutive patients diagnosed with AF (with a new AF diagnosis or already in existing follow-up with a cardiologist) who visited the nurse-led AF clinic for the first time were asked to participate in this trial. Exclusion criteria were patients with cognitive impairment (e.g. severe dementia) and patients who had at least one prior visit with a nurse specialist of the AF clinic. Informed consent was obtained from each patient, and the study protocol conforms to the ethical guidelines of the 1975 Declaration of Helsinki. The study received approval from the ethical committee of the Antwerp University Hospital/University of Antwerp, Jessa Hospital, and Hasselt University.

### 2.2 Study procedures

Demographic data was collected from the medical health records of the patients. The AF clinic offers a structured approach to patients with AF, as described before [14,15,19]. After full evaluation of the patient and his/her profile, tailored education is provided on AF and its treatment in general, and focused on the patient's comorbidity profile [15,20,21]. The patient is given insight in why his/her comorbidity is related to AF (e.g. atrial changes), its course, and its outcomes. This is followed by an individualised treatment plan, with goals set in dialogue with the patient. The whole approach follows a structured path, uniform at both sites. In addition, patients can contact the AF clinic if they have any questions. The first clinic visit lasts approximately one hour, and patient knowledge and progress are regularly evaluated during scheduled follow-ups. Included patients were asked to complete the in-house developed comorbidity assessment questionnaire at three time points: two weeks before the consultation with the AF clinic, less than a week after the consultation, and six months after the consultation with the AF clinic. This questionnaire consisted of three questions for each of nine relevant comorbidities to AF: 1) In general, do you think that this condition affects the occurrence of AF and its consequences? 2) Do you have the condition (whether or not treated)? If yes, 3) Are you being treated for it?. The nine comorbidities were hypertension, overweight, excessive alcohol consumption, physical (in)activity, smoking, low medication adherence, hyperlipidemia, diabetes and sleep disorders. The patients had the opportunity to complete the questionnaire online (via Castor EDC) or by phone interview. The questionnaire was developed by the research team, and nine AF experts (i.e. cardiologists, electrophysiologists, AF nurse specialists, and AF researchers) provided feedback on the content and selected the comorbidities on their importance for AF.

The nurses of the AF clinic used the EHRA-PATHS software to check the presence of 23 comorbidities in these patients before, during and/or after the consultation [17,18]. The software employs a three-step process for assessing comorbidities (and initiating their management): 1) detection triggers (i.e. minimal information needed to assess the possible presence of comorbidity), 2) evaluation/confirmation (i.e. more formal evaluation to further determine whether the comorbidity is present), and 3) a treatment Key Performance Indicator (KPI) (i.e. if any actions have been taken to manage the comorbidity within a six-month timeframe). For this study, only the first two steps were considered. The 3-step approach of the software has been extensively described, together with an example of a care pathway [17,18]. The software was supplemented with an embedded time measurement tool to measure the time needed for completion. This could be split over several sessions. In addition, one to three months after the consultation, the patient had a scheduled telephone contact with the nurse specialist to discuss their comorbidities. The nurses asked patients whether any actions had been taken according to their individualised treatment plan. Additional education and motivation was provided where deemed necessary during the contact. Six months after the consultation, the nurse specialists could contact the patients to obtain additional information to finalise the software (but after the patients had filled out the six-month awareness questionnaire).

### 2.3 Outcomes

The primary outcome of this study was to evaluate awareness of patients with AF for own comorbidities before any AF clinic contact, and whether changes occurred after educational contacts with the AF clinic.

Secondary outcomes were awareness in general about the relevance for AF of the comorbidities, which comorbidities were underestimated or underreported in their contact with the health care team. Also the time needed for a full check of the comorbidities in the EHRA-PATHS software tool, and the total time needed to complete all 23 comorbidity pathways over six months was evaluated, in all and comparing both centres.

### 2.4 Statistical analyses

A sample size calculation was performed to examine whether there are significant differences in the mean number of comorbidities detected by patients with AF over three different time points (within factors repeated measures ANOVA assuming normality). A conventional medium effect size of 0.25, together with an alpha of 0.05 and a power of 0.80 was assumed. The correlation among repeated measures as well as the nonsphericity correction ε remain unchanged and retain values of 0.05 and 1, respectively. The output of the power analysis indicated a sample size of at least 28 participants. Taking into account a possible dropout rate of 25 %, this led to 38 patients. To have a balanced representation of both clinics, we aimed for 76 patients in total.

The collected data were analysed using the statistical program SPSS version 29.0 or RStudio 2023.12.1. The normality check of the data was done using the Shapiro-Wilk test. The mean ± SD was used to report all normally distributed continuous data, and the median and interquartile range (IQR) were used to report non-normally distributed continuous data. Categorical variables were demonstrated as numbers (percentages). The Friedman test was used to evaluate the difference in three time points, whether patients were aware that the comorbidities could affect their AF. Moreover, the difference in time between both centres was analysed using the Mann-Whitney *U* test. In addition, the Spearman’s correlation analysis was used to examine the association between the number of present comorbidities and the needed time. The generalised mixed model was used to analyse the changes in comorbidity awareness over time. The sub-analysis between two groups (i.e. new AF or AF with existing follow-up) was analysed using the linear mixed model. Regarding the timing, outlier analysis was performed by excluding the timing of comorbidities that were completed multiple times. Results with a p-value < 0.05 were accepted as statistically significant.

## 3 Results

### 3.1 Patient characteristics

**Fig. 1:**
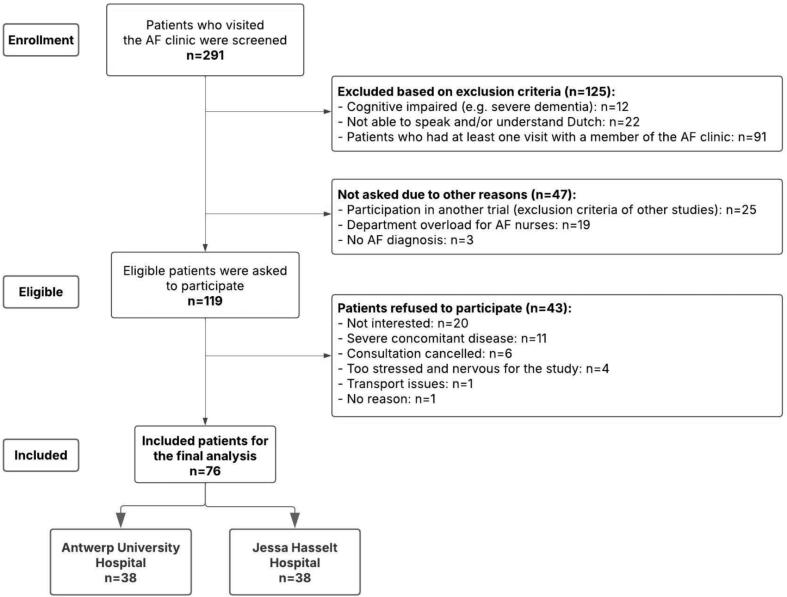
Flowchart of the screened and included patients. AF: Atrial fibrillation.

In total, 291 patients were screened for this study (Fig. 1). Ninety-one patients (31.3 %) had to be excluded because they had at least one prior visit to the AF clinic. Fig. 1 indicates other reasons for exclusion. A total of 76 patients were included in the study, 38 patients in each centre. Table 1 shows the demographic characteristics of all patients. The included patients had a mean age of 68.3 ± 10.3 years, of which 68.4 % were men. Most of the patients had persistent AF (39.5 %), followed by paroxysmal (30.3 %), long-standing persistent AF (28.9 %), and novo AF (1.3 %). Moreover, the patients had a median AF duration of 5.5 (1–71) months at inclusion time. A total of 36 patients (47.4 %) were diagnosed with AF in ≤ six months, while 36 patients (47.4 %) had been diagnosed with AF more than six months (AF duration was not reported for four patients). More patients completed secondary school (43.4 %), followed by college (38.2 %), primary school (9.2 %) and university (9.2 %).

**Table 1:** Demographic table with baseline characteristics of included AF patients (n = 76).

	Total (n = 76)
**Age, yrs, mean ± SD**	68.28 ± 10.34
**Male, n (%)**	52 (68.4 %)
**Highest educational degree n (%)**	
Primary school	7 (9.2 %)
Secondary school	33 (43.4 %)
College	29 (38.2 %)
University	7 (9.2 %)
**Risk factors and comorbidities based on medical record at baseline, n (%)**	
Hypertension	40 (52.6 %)
Cardiac family history	34 (44.7 %)
Vascular disease	16 (21.1 %)
Heart failure	15 (19.7 %)
Prior stroke, TIA, thromboembolism	6 (7.9 %)
Diabetes	5 (6.6 %)
Abnormal renal function	5 (6.6 %)
Abnormal liver function	3 (3.9 %)
Bleeding	3 (3.9 %)
**mEHRA score, n (%)**	
1	11 (14.5 %)
2a	25 (32.9 %)
2b	32 (42.1 %)
3	8 (10.5 %)
4	0 (0.0 %)
**CHA_2_DS_2_-VA score, median (IQR)**	2 (1–4)
**HAS-BLED score, median (IQR)**	1 (1–2)
**Medication, n (%)**	
Rate control	25 (32.9 %)
Rhythm control	46 (60.5 %)
**AF related parameters**	
AF duration, months, median (IQR)^a^	5.5 (1.0–71.0)
AF duration ≤ 6 months, n (%)	36 (47.4 %)
AF duration > 6 months, n (%)	36 (47.4 %)
Type of AF, n (%)	
First diagnosed AF	1 (1.3 %)
Paroxysmal AF	23 (30.3 %)
Persistent AF	30 (39.5 %)
Long-standing persistent AF	22 (28.9 %)
Patients with prior ablation, n (%)	20 (26.3 %)
Number of ablations, (median (IQR))	1 (1–2)
Type of ablation, n (%)	
Flutter ablation	3 (3.9 %)
PVI cryo	5 (6.6 %)
PVI electroporation	4 (5.3 %)
PVI laser PVI radio	1 (1.3 %) 12 (15.8 %)
**Echocardiographic measurements**	
Ejection fraction, %, median (IQR)^b^	60.0 (55.0–61.0)
LAVI > 34 ml/m^2^, n (%)^c^	
LAVI diameter (ml/m^2^), (median (IQR))^d^	48 (63.2 %)
Mitral regurgitation, n (%)^e^	46.5 (38.3–51.5)
Grade I	50 (89.3 %)
Grade II	4 (7.1 %)
Grade III	2 (3.6 %)
Grade IV	0 (0.0 %)

Normally distributed values are demonstrated as mean ± SD, non-normally distributed values are as median (IQR) and categorical values as n (%).

*Comparison between the two centres (p < 0.05).

AF: Atrial fibrillation; IQR: Interquartile range; LAVI: Left atrial volume index; mEHRA: modified European Heart Rhythm Association score; PVI: Pulmonary vein isolation; TIA: transient ischemic attack; SD: Standard deviation; CHA_2_DS_2_-VA score (Congestive heart failure, age ≥ 75 years, Diabetes Mellitus, prior stroke/TIA/thromboembolism, Vascular disease, age 65–74 years and sex category). HAS-BLED score (Hypertension, abnormal renal and liver function, prior stroke, Bleeding, Labile INR, elderly and drugs or alcohol).

a 4 unknown.

b 9 unknown.

c 8 unknown.

d 48 unknown.

e 20 unknown.

Using the software tool, patients had a median of 4.0 (3.0–5.5) comorbidities. The three most common comorbidities in this included AF population were hyperlipidemia (present in 77.6 %, 59 of 76), overweight (present in 61.8 %, 47 of 76), and hypertension (present in 60.5 %, 46 of 76).

### 3.2 Comorbidity awareness of patients

**Fig. 2:**
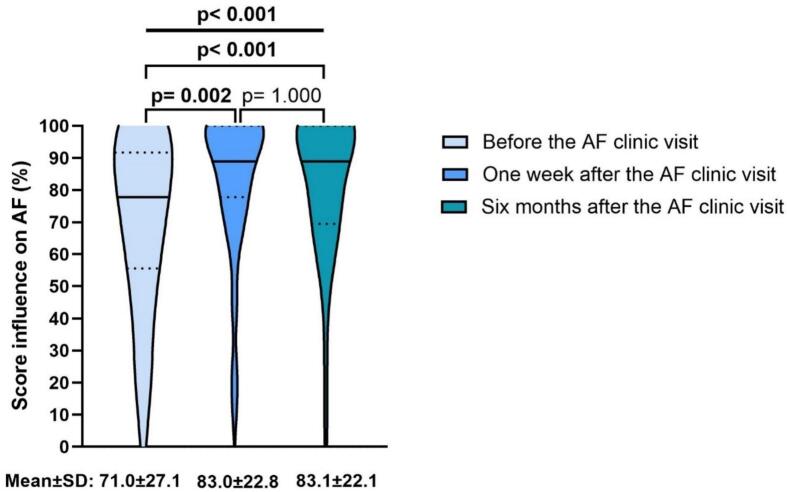
Patients’ scores on whether nine different comorbidities could affect the occurrence of AF and its consequences. Before, one week, and six months after the first AF clinic visit. Data are shown as violin plots with median and interquartile range. The mean ± SD score is also mentioned below. AF: Atrial fibrillation; SD: Standard deviation. Friedman test.

The first question of the comorbidity awareness questionnaire was “In general, do you think that this condition affects the occurrence of AF and its consequences?”. Fig. 2 demonstrates that patients were more aware that the nine comorbidities could affect AF six months after the first visit with the AF nurse specialist compared to before the first AF clinic visit (median before: 77.8 % vs. after one week: 88.9 % vs. after six months: 88.9 %; p < 0.001).

The next question was “Do you have the condition (whether or not treated)?”. The patients underestimated the presence comorbidities as “present-in-me” compared to the median of 4 by the software tool, without any significant change over the different time points (before: 2 [[1], [2], [3]] vs. after one week: 3 [[2], [3], [4]] vs. after six months: 3 [2,3]; p = 0.092).

**Fig. 3:**
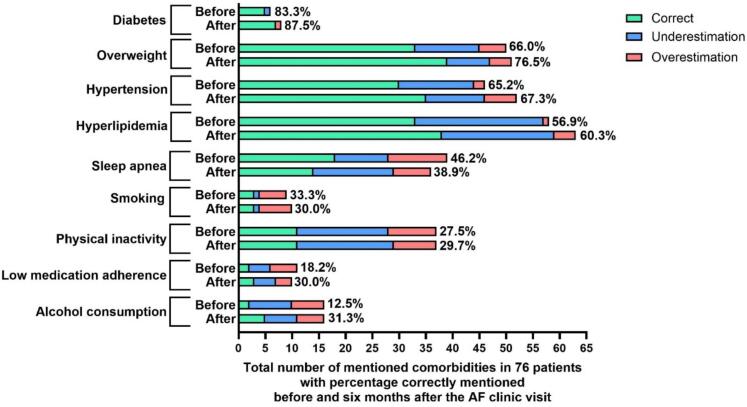
The number of times a specific comorbidity was mentioned correctly, underestimated, or overestimated by patients compared to the AF nurse specialist before and after six months after the first contact with the AF clinic. Green means the patient and the nurse specialist at the AF clinic report the same comorbidity. Blue means the patients underestimated the comorbidity compared to the nurse specialist at the AF clinic, and red means the patients overestimated the comorbidity compared to the nurse specialist. In addition, the percentages correctly mentioned comorbidities are shown. AF: Atrial fibrillation.

Before the first AF clinic visit, patients were best aware of having diabetes (83.3 %, 5 of 6), while only 12.5 % (2 of 16) were aware of excess alcohol consumption (Fig. 3). Alcohol consumption was the most underestimated comorbidity (50.0 %, 8 of 16), followed by physical inactivity (42.5 %, 17 of 40). Intriguingly some comorbidities were overestimated compared to the assessment by the AF nurse, likely due to more patients reported on the questionnaire in private than during the personal contact: this was seen for smoking in 55.6 % of the smokers (5 of 9), 37.5 % of alcohol overuse (6 of 16), 28.2 % of sleep apnea (11 of 39), and 22.5 % of physical inactivity (9 of 40).

When re-evaluated at six months, while there was a slightly (but not significant) better correct self-awareness for the most common comorbidities, i.e. hyperlipidemia (56.9 % to 60.3 %; p = 0.200), hypertension (65.2 % to 67.3 %; p = 0.150), and overweight (66.0 % to 76.5 %; p = 0.560), the overall picture of insufficient awareness remained (p = 0.099) (Fig. 3). Moreover, some comorbidities remained ‘overestimated’. Even shortly after the first clinic visit with education, the awareness pattern was not impacted significantly (p = 0.456) (Supplementary Fig. 1).

**Fig. 4:**
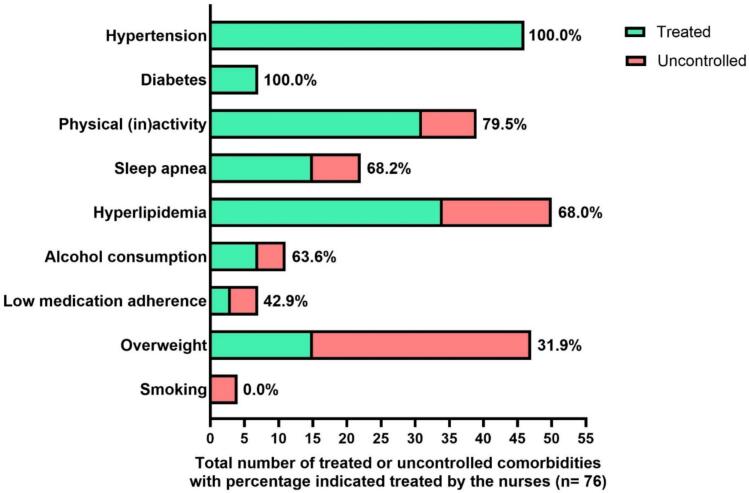
The number of times a specific comorbidity was mentioned treated or uncontrolled by the AF nurse specialist in the software tool at the end of the study. Green means the nurse specialist at the AF clinic reports the comorbidity is treated. Red means the nurse specialist at the AF clinic reports the comorbidity is uncontrolled. In addition, the percentages indicated treated comorbidities are shown.

At the six-month time point, management initiation for the present comorbidities was evaluated using the EHRA-PATHS software. Fig. 4 shows that all patients with hypertension and diabetes were on treatment, while 68.1 % of patients with overweight (32 of 47) had not taken the steps that were discussed with them during the initial visit or during the ensuing telephone contact with the AF nurse. Moreover, some patients also did not follow the necessary actions for their other comorbidities: Low medication adherence (57.1 %; 4 of 7), alcohol consumption (36.4 %; 4 of 11), hyperlipidemia (32.0 %; 16 of 50), sleep apnea (31.8 %; 7 of 22), and physical inactivity (20.5 %; 8 of 39).

Sub-analysis on patients diagnosed with AF ≤ six months or AF > six months shows no significant differences between both groups, before (p = 0.153), after one week (p = 0.401) and six months after the first AF clinic visit (p = 0.800) (Supplementary Fig. 2).

### 3.3 Time needed for comorbidity assessment

**Fig. 5:**
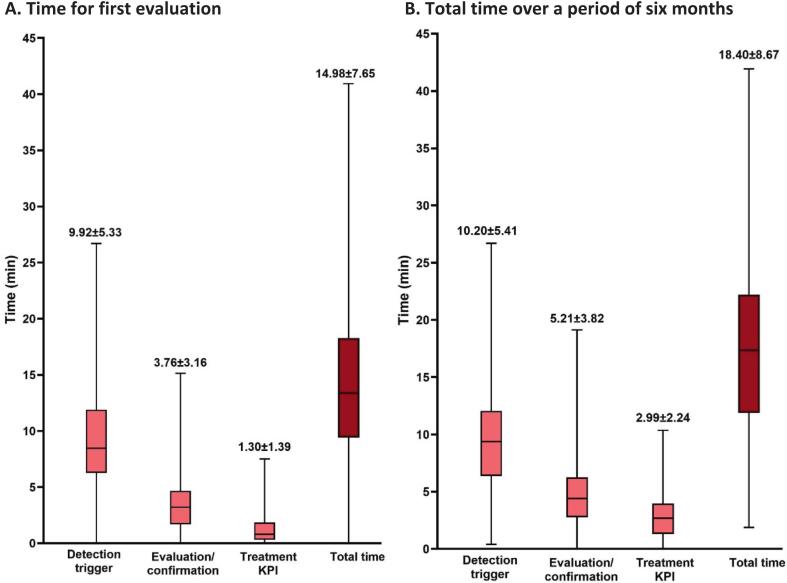
The time needed for the first evaluation (A) and the total time over six months (B) for completion of all 23 comorbidity care pathways (n = 76). Data are shown as median and interquartile range. The average time is also mentioned (mean ± standard deviation). KPI = Key Performance Indicator.

Fig. 5 shows the time needed for the full assessment of the 23 comorbidities using the EHRA-PATHS software tool, at the first clinic visit (panel A), and within the full six-month time frame (panel B), during which all 3 steps for all comorbidities need to be filled out. The initial evaluation at the first visit required 14.98 ± 7.65 min, leading to the detection of a median of 4 comorbidities. Later completion of the confirmation step (step 2) and of the treatment initiation step (step 3) did not add much time, resulting in a total of 18.40 ± 8.67 min on average per patient.

The mean time needed for evaluation was significantly longer in one centre compared to the other, both for the first evaluation and over the six-month period (Supplementary Fig. 3). The evaluation of the detection triggers (step 1) made the difference, likely due to a different practice in which one centre started filling out the software before the first contact (looking at the medical records) and completed the software during and after that first contact, required overall more time than the centre that filled out the software only after the first clinic visit of the patient.

**Fig. 6:**
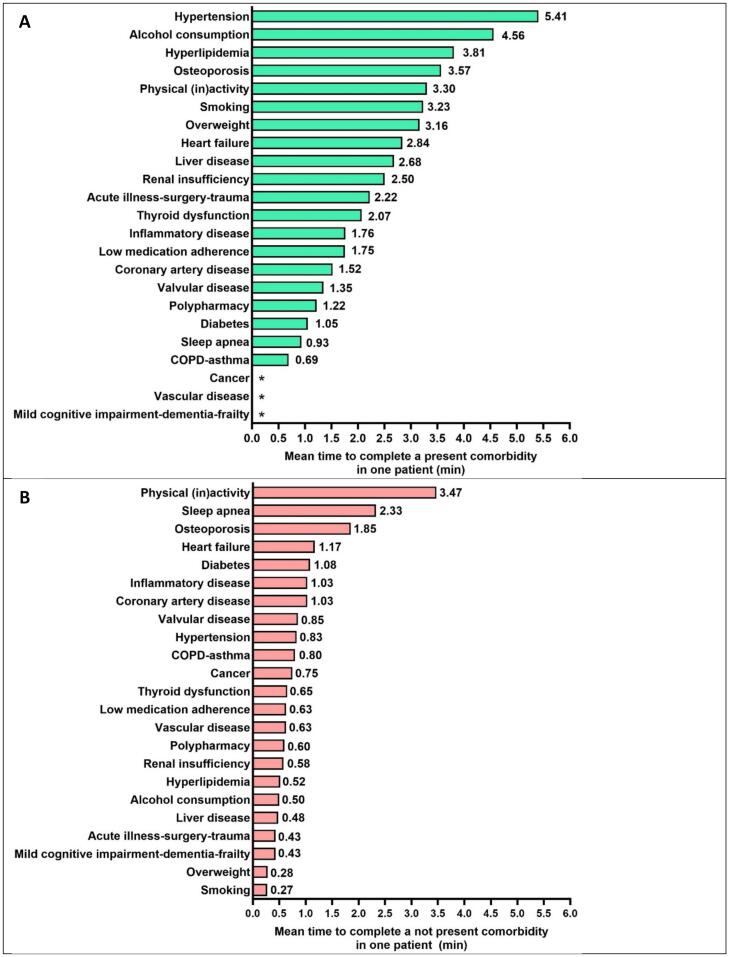
Total time needed for present comorbidity (A) and excluded comorbidity (B) for completion of all steps (n = 76). * No timing was recorded for cancer, vascular disease and mild cognitive impairment-dementia-frailty because these comorbidities were not present in the included AF population. COPD: Chronic obstructive pulmonary disease.

The number of present comorbidities showed a weakly positive but not-significant correlation with the time for completion (r = 0.155, p = 0.180), as shown in Supplementary Fig. 4. This may be related to the unequal time needed in the software for evaluation of different comorbidities. For present comorbidities, the most time was required for hypertension (5.41 min), alcohol consumption (4.56 min) and hyperlipidemia (3.81 min), while COPD-asthma (0.69 min) and sleep apnea (0.93 min) were the fastest to complete (Fig. 6A). Conversely, to exclude a comorbidity, most time was needed in the software for physical (in)activity (3.47 min) and sleep apnea (2.33 min), while smoking (0.27 min) and overweight (0.28 min) required the least time to exclude (Fig. 6B). No timing was recorded for cancer, vascular disease and mild cognitive impairment-dementia-frailty because these comorbidities were not present in the included AF population (Fig. 6A).

## 4 Discussion

This study showed that comorbidity awareness of patients with AF on the relevance of comorbidities in general was reasonable at the outset, and could be further improved by contacts with the AF clinic. However, the awareness of personal comorbidities was suboptimal, with frequent underestimation that proved hard to correct despite an in-person visit and a telephone follow-up contact after one to three months. We also showed that systematic and complete comorbidity evaluation at the AF clinic with the EHRA-PATHS software can be done within a reasonable time frame of 15–20 min.

### 4.1 Comorbidity awareness of patients, and improving it

Management of comorbidities is the first focus of the 2024 ESC guidelines for AF management [10], as made clear from the acronym CARE-AF. Integrated care means that patients are co-owners of their care, which starts with awareness about their comorbidities. We have shown suboptimal comorbidity awareness, with little impact of a single in-person session and additional phone consultation from the AF clinic. It is clear that more educational efforts are needed to improve patients’ awareness and start a joint path towards comorbidity management.

Comorbidities that already have structured follow-up (e.g. diabetes) clearly showed the best awareness. Most of these patients were also effectively managed, as could be seen in Fig. 4. Intriguingly, some comorbidities were overestimated. For overweight, this may have to do with the fact that the EHRA-PATHS software uses a criterion of a BMI > 27 kg/m^2^. We noted that a patient with a BMI of 25.7 kg/m^2^ at inclusion, which further increased to 26.6 kg/m^2^ after six months, self-reported correctly ‘overweight’ or weight increase, while the software did not retain the comorbidity. This could be addressed in the future. Other comorbidities with ‘overestimation’, like alcohol consumption or smoking can be explained by the fact that patients are more honest when filling out the questionnaire on their own, while being more reticent in the interview with the nurse during filling out the software. This is also the reason why fewer smokers are seen in Fig. 4 (n = 4) compared to Fig. 3 (n = 10). This could be addressed by adding prior self-reporting to nurse-guided software completion. Physical inactivity remains a difficult comorbidity to tackle, starting with the fact that 42.5 % of patients (17 of 40) underestimated their inactivity before the first visit, and even 48.6 % (18 of 37) after six months. If inactivity is not recognised as a problem by the patient, it will remain very hard to modify it, let alone to get to the guideline goal of ≥ 150 min per week of moderate physical activity [10,22].

Fig. 4 was an essential aspect of whether the present comorbidities in the software are managed well. The management of overweight (68.1 %) and smoking (0.0 %) was insufficient six months after the AF clinic. All patients who were overweight were asked if they measured and recorded their weight at least once a week, but 62.5 % of patients (20 of 32) did not do so. Patients with a BMI ≥ 30 kg/m^2^ had to be referred (according to the software tool) to a dietitian or formal weight loss program, but this referral was not the case for 71.4 % of those patients (15 of 21). Similarly, no referrals to a tobacconist or structured smoking cessation counselling were provided for smokers. Medication adherence was also not addressed in 57.1 % of patients (4 of 7), with three patients who had no scheduled follow-up and one patient having an INR time in therapeutic range of just 20 %. These findings indicate that step 3 in the software requires more attention as it is the goal to manage comorbidities! However, it may be too strict for some patients and may require optimisation for future use.

Although our results highlight the lack of comorbidity awareness and management, it is important to keep in mind that patient behaviour may not be changed by creating awareness and referral alone. From the patient’s perspective, lifestyle modifications and adherence to referrals are frequently challenged by barriers such as a lack of confidence in their ability to change, competing daily priorities, or a lack of urgency to take action. To effectively address these challenges, approaches like motivational interviewing, individualised goal setting, and shared decision-making may be necessary to complement systematic mapping and referral strategies.

Prior studies have examined the knowledge of patients with AF about their condition and its management, and how that awareness could be improved. The AF-EduCare trial showed that by use of a self-care questionnaire with 14 questions related to AF risk factors, patients often underestimated their own AF risk factors [15]. It showed, however, that intensive and targeted education of patients with AF enhanced their knowledge of AF-related topics, raised their awareness about comorbidities, and resulted in higher medication adherence [14]. It is clear that more is needed than an initial contact and a 1–3 month follow-up phone contact.

### 4.2 Time investment for full comorbidity mapping in daily practice

As this is the first clinical study examining the use of a software tool to systematically evaluate comorbidities in patients with AF, and to measure the time needed to complete all 23 comorbidity care pathways, it is not possible to compare with prior literature. The mean time needed for the first evaluation was just under 15 min, which increased to 18.40 min over a total of six months. This time investment seems acceptable for the systematic evaluation of patients who will require long-term follow-up of their chronic arrhythmic condition.

The fact that the detection trigger required most time to complete is understandable, given that the 23 comorbidities needed a first check on presence or exclusion. The evaluation/confirmation and treatment KPI steps (steps 2 and 3) were only required to be completed if a comorbidity was suspected or confirmed at initial evaluation. The fact that there was a significant difference in time needed for both centres may be due to a different learning curve (although it was new to both centres), or to forgetting to close the software window when answering telephone calls leading to extended time without using the software (although the outliers were analysed and removed). It may however also teach us that there may be ‘best practices’ on how the software tool: it may be preferable to first see the patient, ask the required questions, and then fill out the software, as was done in one centre, rather than starting with a prior evaluation of the chart to partially fill-out the comorbidity pathways as was done in the other centre. The analysis of time-investment for the respective comorbidities, as shown in Fig. 6, can also be used for further optimisation of the software. E.g. the long time for alcohol consumption evaluation can be explained by the fact that a questionnaire needs to be completed. And although hypertension is in essence a simple comorbidity care pathway, the long durations recorded for its completion could be due to the fact that nurses may have measured blood pressure, or accessed the medical health records for blood pressure values, without closing the software window. Another explanation could be that hypertension was the first comorbidity in the software tool, and it could be that the nurses left the screen open longer while also reviewing other comorbidities. All in all, there is good hope that the time investment for using the software can be shortened by optimising the care-pathway evaluation. This will lead to more acceptance for its use in busy practices, and as a guarantee that more patients may be offered a systematic comorbidity evaluation which is crucial for their optimal management.

### 4.3 Study limitations

The study had a rather small sample size and a short follow-up period of only six months, which does not allow assessment of the clinical impact of the comorbidity evaluation. However, that was not the aim of this trial, since the clinical trial that is part of the EHRA-PATHS project (ClinicalTrials.gov NCT05773768) is set up to do just that. Another limitation is that the comorbidity questionnaire was developed in-house and has not been formally validated. Patients were not directly involved in the development of the questionnaire, which may have limited the ability to capture their perspectives, or may have led to their suboptimal understanding of the questions themselves. Future research is needed to validate the questionnaire further. The embedded time registration tool records the full duration that care pathway windows are open and does not close automatically when the AF nurses do not use the software. Although we instructed our nurses very specifically to always close windows (e.g. when they received an incoming phone call), this cannot be excluded. Outlier analysis however did not show clinically unexplainable long-time durations of open windows. Finally, a different ability to learn new software by different AF nurses may have contributed to timing differences in a relatively small study.

### 4.4 Future perspectives

A single in-person education session and a 1–3 month phone follow-up contact is insufficient to significantly impact patients’ awareness on their comorbidities. Therefore, more thorough educational interventions are required [14,15]. Improvement of some of the software tool’s care pathways, education of nurses using it, a checklist with all needed parameters in the software will undoubtedly further reduce the completion time, which is essential for daily clinical practice. We hope that our efforts will lead to a standardised, efficient, systematic clinical approach for comorbidity assessment and management of patients with AF.

## 5 Conclusions

Comorbidity awareness among patients with AF is suboptimal and more educational efforts than a single session plus follow-up phone consult are needed to improve it. A systematic and complete comorbidity evaluation at the AF clinic with the EHRA-PATHS software can be done within a reasonable timeframe now, with possibilities for further optimisation. This will lead to more acceptance for its use in busy practices, and as a guarantee that more patients may be offered a systematic comorbidity evaluation and education which is crucial for their optimal management.

## 6 Grant support

The EHRA-PATHS project has received funding from the European Union’s Horizon 2020 research and innovation programme under grant agreement No 945260.

## CRediT authorship contribution statement

**Rana Önder:** Writing – review & editing, Writing – original draft, Visualization, Validation, Supervision, Resources, Project administration, Methodology, Investigation, Formal analysis, Data curation, Conceptualization. **Lien Desteghe:** Writing – review & editing, Supervision, Conceptualization, Project administration. **Johan Vijgen:** Writing – review & editing, Supervision, Conceptualization, Project administration. **Hein Heidbuchel:** Writing – review & editing, Supervision, Conceptualization, Project administration.

## Declaration of competing interest

The authors declare the following financial interests/personal relationships which may be considered as potential competing interests: R.Ö., L.D. and J.V. report no relationships that could be construed as a conflict of interest. H.H. receive personal lecture and consultancy fees from, Biotronik, Daiichi-Sankyo, Downtown Europe, IZIDOK, European Society of Cardiology, and Viatris Pharmaceuticals Inc. He received unconditional research grants through the University of Antwerp and/or the University of Hasselt from Abbott, Bayer, Boehringer-Ingelheim, Biosense-Webster, Boston-Scientific, Daicchi-Sankyo, Viatris Pharmaceuticals Inc, Novo Nordisk, Novartis, and Pfizer-BMS, all outside the scope of this work.
