# Supplementary material for: Assessment of comorbidity awareness in patients with atrial fibrillation: The ACAPAF study
Source: Int J Cardiol Heart Vasc. 2025 Oct 2;61:101813. doi: 10.1016/j.ijcha.2025.101813 (PMC12522701; doi:10.1016/j.ijcha.2025.101813)
Supplement: Supplementary Data 1 [file mmc1.docx]

# Supplementary figures

**Supplementary Figure 1**

**The number of times a specific comorbidity was mentioned correctly, underestimated, or overestimated by patients compared to the AF nurse specialist before, one week after, and six months after the first contact with the AF clinic.**


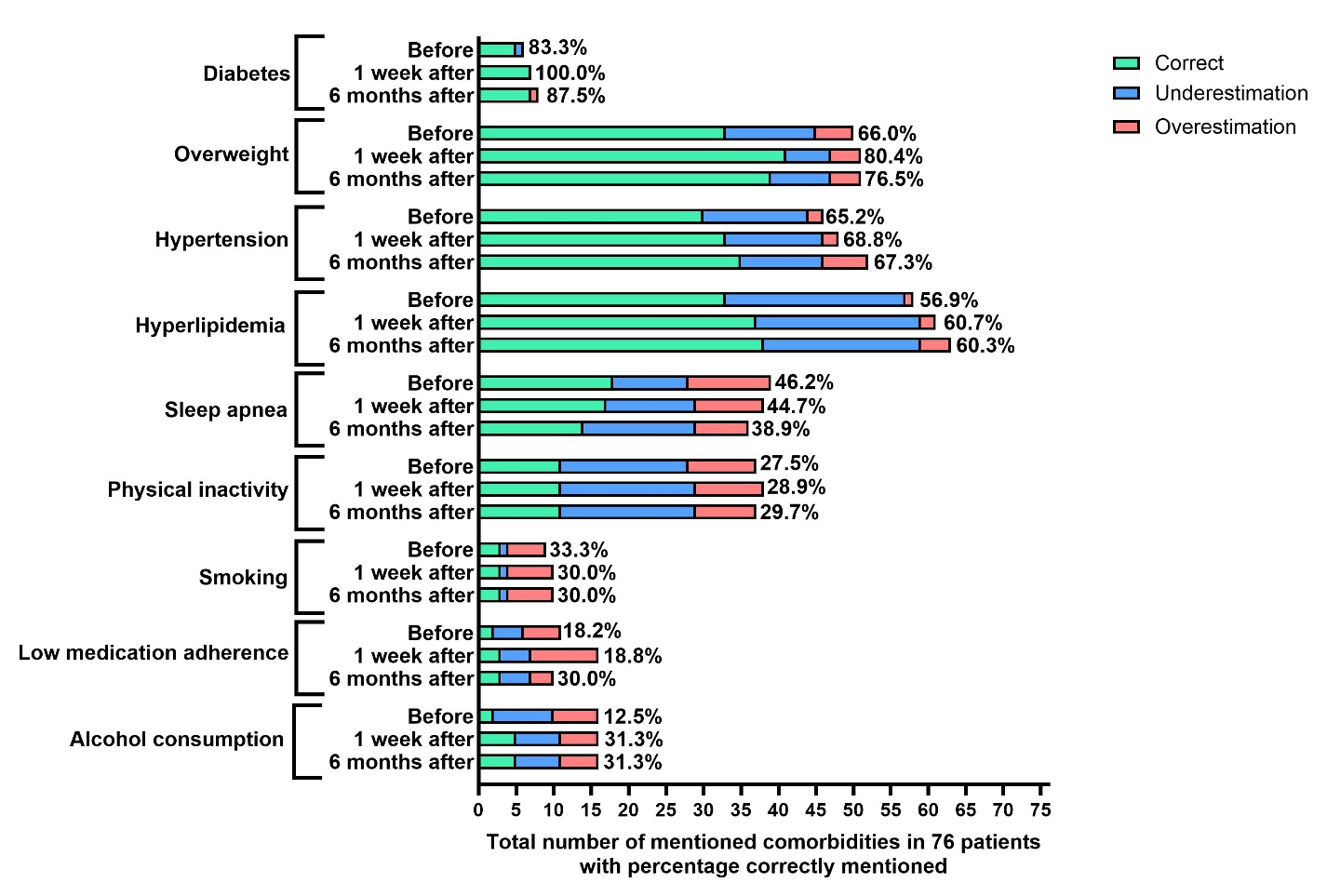


Green means the patient and the nurse specialist at the AF clinic report the same comorbidity. Blue means the patients underestimated the comorbidity compared to the nurse specialist at the AF clinic, and red means the patients overestimated the comorbidity compared to the nurse specialist. In addition, the percentages correctly mentioned comorbidities are shown.

**Supplementary Figure 2**

**The number of times a specific comorbidity was mentioned correctly, underestimated, or overestimated by (A-C) patients diagnosed with AF ≤ six months and (D-F) patients diagnosed with AF > six months compared to the AF nurse specialist.**

| **Patients diagnosed with AF ≤ six months (A-C)**  **A.** **Comparison before the first contact with the AF clinic**  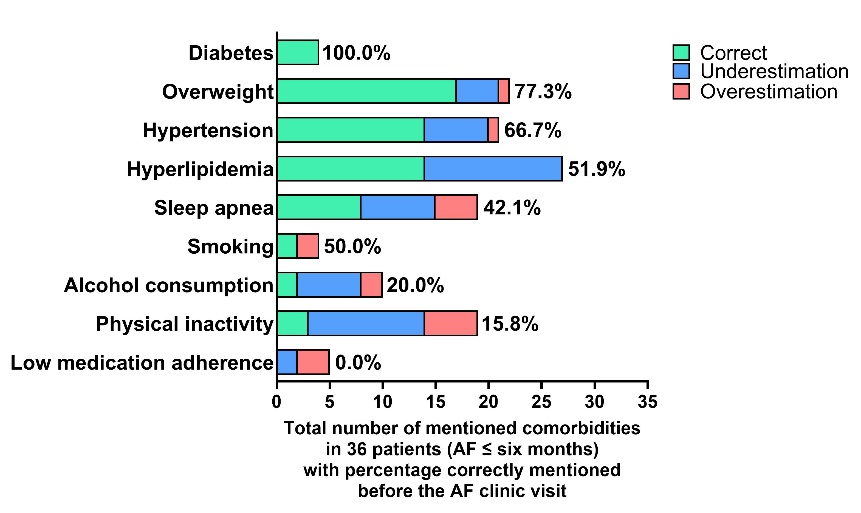 | **Patients diagnosed with AF > six months (D-F)**  **D. Comparison before the first contact with the AF clinic**  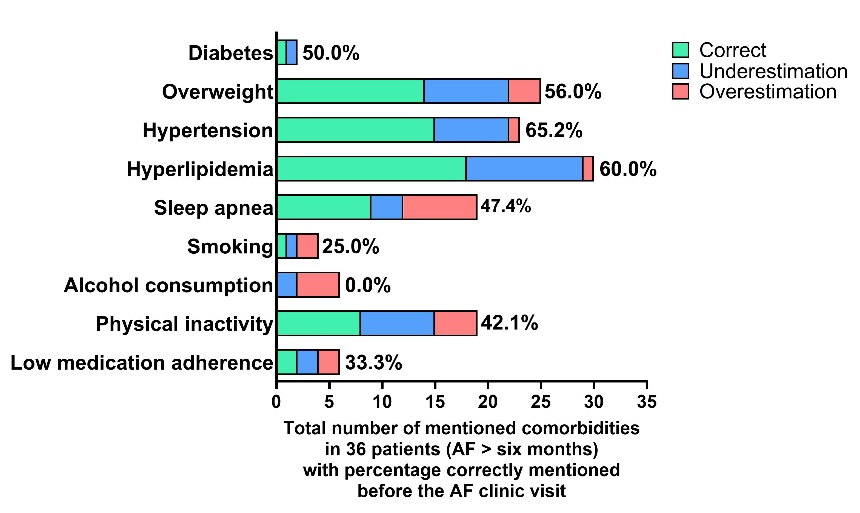 |
| --- | --- |
| **B. Comparison one week after the first contact with the AF clinic**  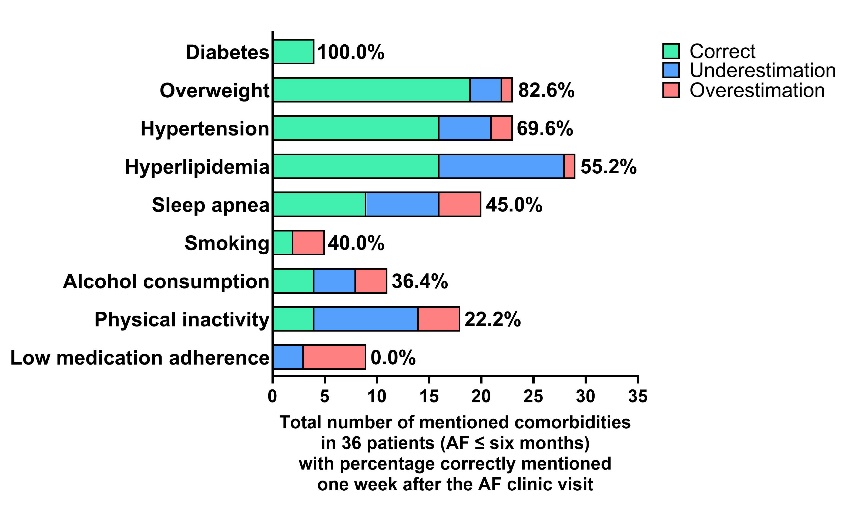 | **E. Comparison one week after the first contact with the AF clinic**  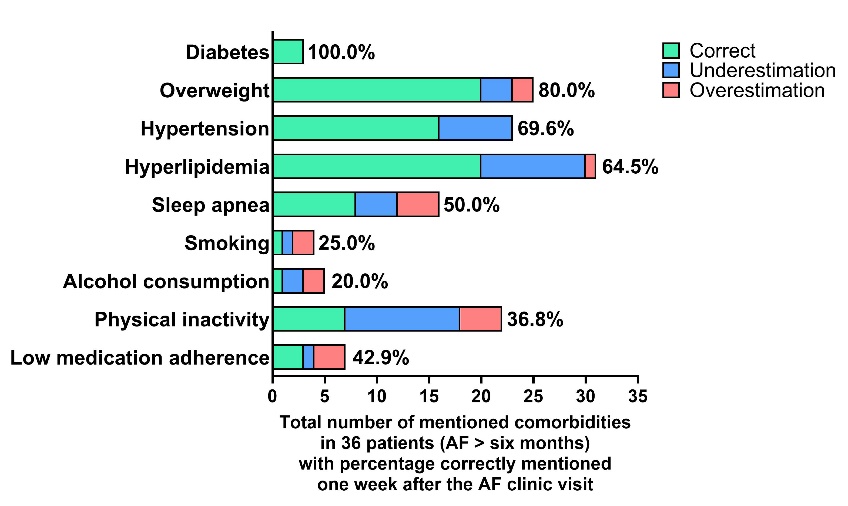 |
| **C. Comparison six months after the first contact with the AF clinic**  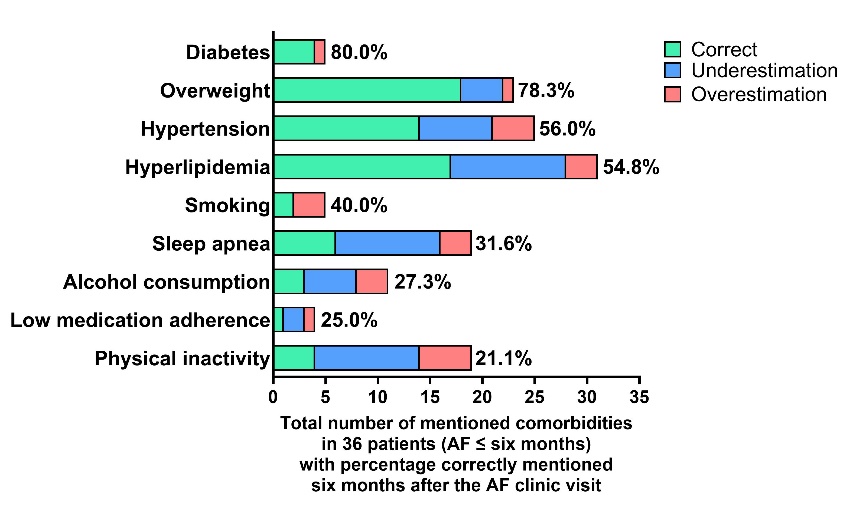 | **F. Comparison six months after the first contact with the AF clinic**  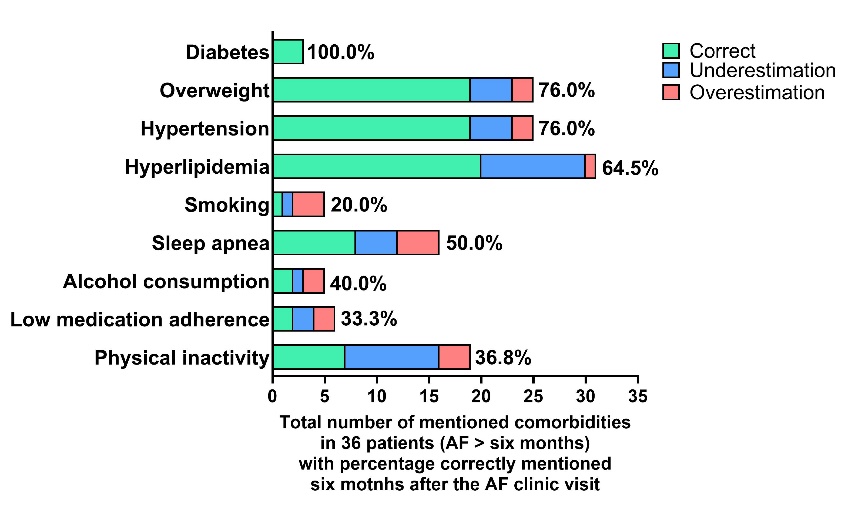 |

Green means the patient and the nurse specialist at the AF clinic report the same comorbidity. Blue means the patients underestimated the comorbidity compared to the nurse specialist at the AF clinic, and red means the patients overestimated the comorbidity compared to the nurse specialist. In addition, the percentages correctly mentioned comorbidities are shown.

**Supplementary Figure 3**

**Comparison of the time needed for the first evaluation (A) and total time over six months (B) for completion of all 23 comorbidity care pathways in both centres (n=38 and n=38).**

| 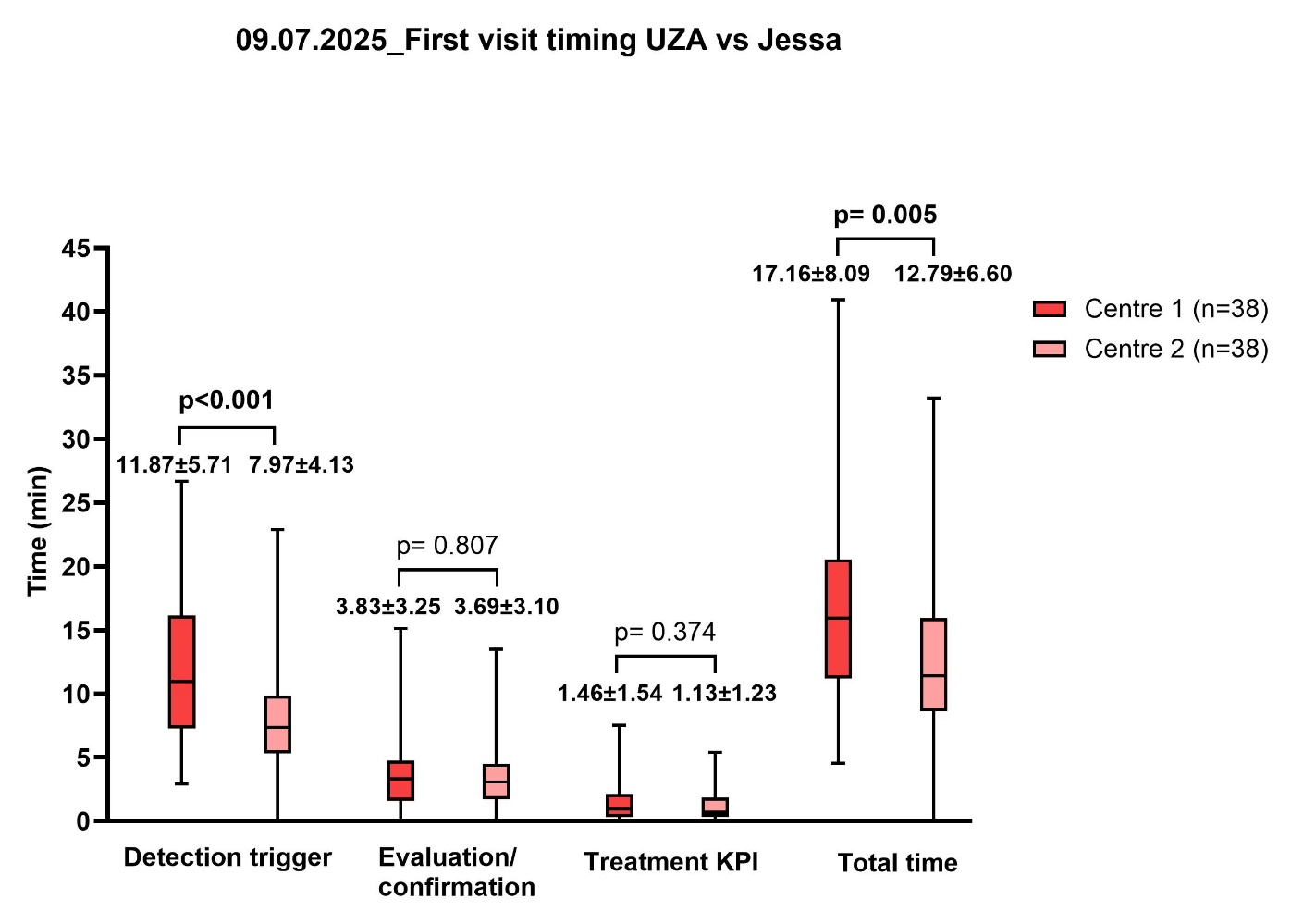  **A. Time for first evaluation** |
| --- |
| 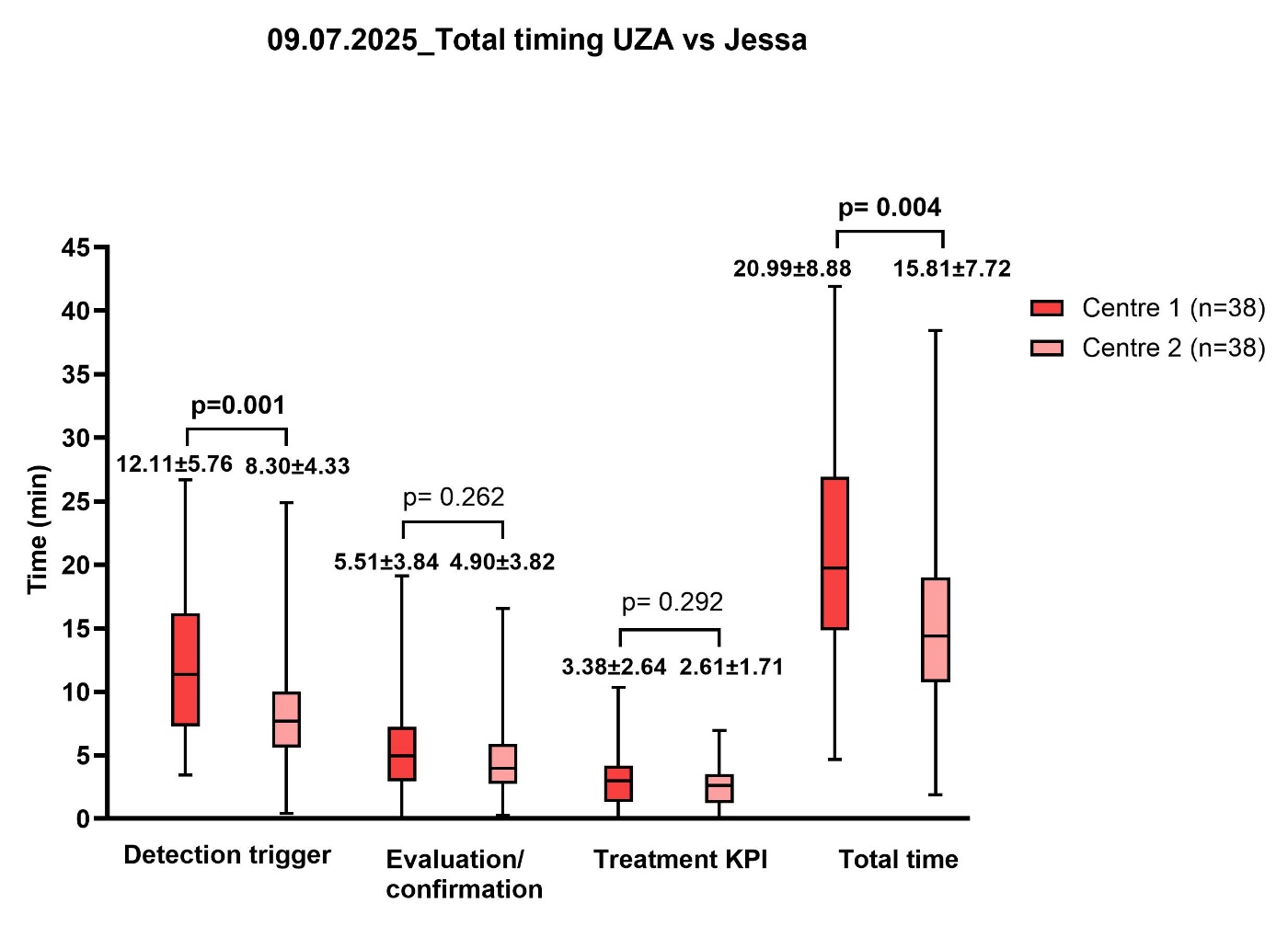  **B. Total time over a period of six months** |

Data are shown as median and interquartile range. The average time is also mentioned (mean±standard deviation). The Mann-Whitney U test. KPI= Key Performance Indicator

**Supplementary Figure 4**

**Correlation between the total time for completion and the number of present comorbidities (n= 76)**


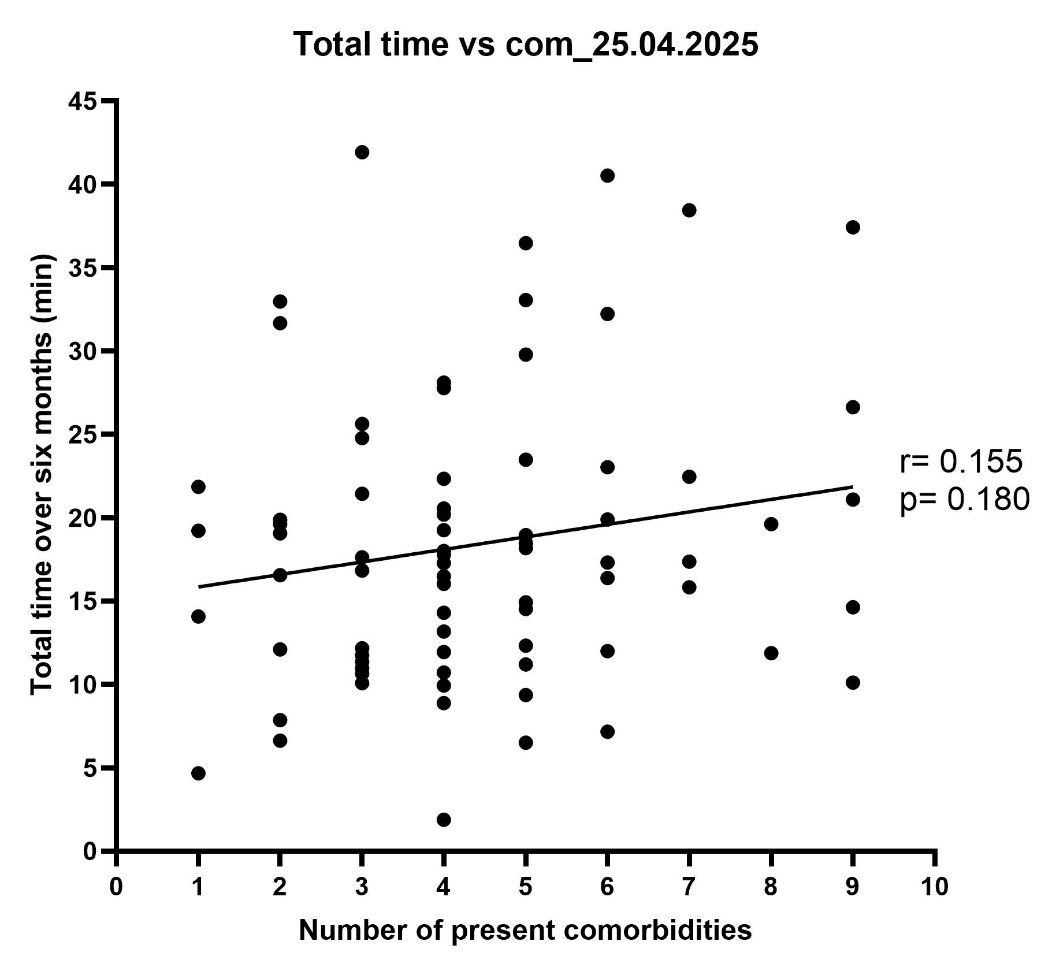


r= Correlation coefficient. Spearman’s rho correlation.
